# Hypermetabolic Hurthle Cell Adenoma on ^18^F-FDG PET/CT

**DOI:** 10.4274/mirt.49469

**Published:** 2018-06-07

**Authors:** Aamna Hassan, Saima Riaz, Amna Asif

**Affiliations:** 1 Shaukat Khanum Memorial Cancer Hospital and Research Centre, Department of Nuclear Medicine, Lahore, Pakistan; 2 Shaukat Khanum Memorial Cancer Hospital and Research Centre, Department of Pathology, Lahore, Pakistan

**Keywords:** Thyroid, Incidentaloma, 18F-FDG PET/CT, Hurthle cell adenoma

## Abstract

Thyroid incidentalomas are frequently reported on ^18^F-FDG PET/CT scan. High risk of malignancy is thought to be associated with increased metabolic activity and high standardized uptake value. Likewise, thyroid nodules with focal FDG avidity have a higher potential to be malignant. However, some benign nodules such as follicular and Hurthle cell adenomas can also present with focal hypermetabolic activity. We report a case of a 59-year-old lady diagnosed with gastric carcinoma, who had a hypermetabolic thyroid nodule on FDG PET/CT scan. Despite the complex texture of the nodule and intense focal avidity, the histopathology was consistent with Hurtle cell adenoma.

## Abstract (tr)

^18^F-FDG PET/BT görüntülemede tiroid insidentalomalarına sık rastlanır. Yüksek malignite riski artmış metabolik aktivite ve yüksek standart tutulum değeri ile ilişkilendirilmektedir. Fokal FDG tutulumu olan tiroid nodüllerinde malignite olasılığı benzer şekilde artmaktadır. Ne var ki, foliküler ve Hurthle hücreli adenom gibi bazı benign nodüller fokal hipermetabolik aktivite gösterebilir. Bu yazıda gastrik adenokarsinom tanısı almış ve FDG PET/BT’de hipermetabolik tiroid nodülü saptanan 59 yaşında bir kadın hasta sunulmaktadır. Nodülün sert yapısı ve yoğun fokal tutuluma rağmen histopatoloji değerlendirmesi Hurthle hücreli adenom ile uyumlu idi.

## Figures and Tables

**Figure 1:**
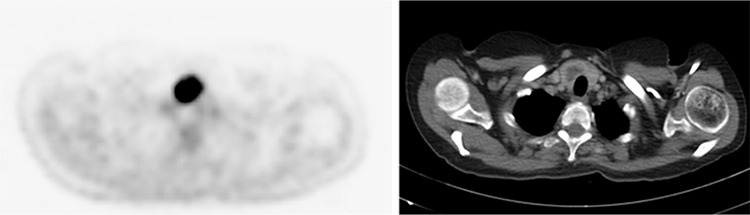
A 59-year-old female patient with a diagnosis of gastric carcinoma was referred for staging ^18^F-FDG PET/CT scan. Imaging was performed 60 minutes after a dose of 294 MBq, on an integrated 16-slice PET/CT scanner, with scanning from the vertex to the mid-thigh. ^18^F-FDG PET/CT scan axial views of the lower neck showed a hypermetabolic, heterogeneously enhancing right thyroid nodule with low attenuation [2.7 cm, standardized uptake value (SUV) 17.5]. The left thyroid lobe was unremarkable both radiologically and metabolically. Ultrasound correlation and targeted biopsy of the right thyroid nodule was recommended. The patient was clinically and biochemically euthyroid. Fine needle aspiration cytology was consistent with follicular neoplasm, Bethesda category IV. Thereafter, the patient underwent partial thyroidectomy.

**Figure 2:**
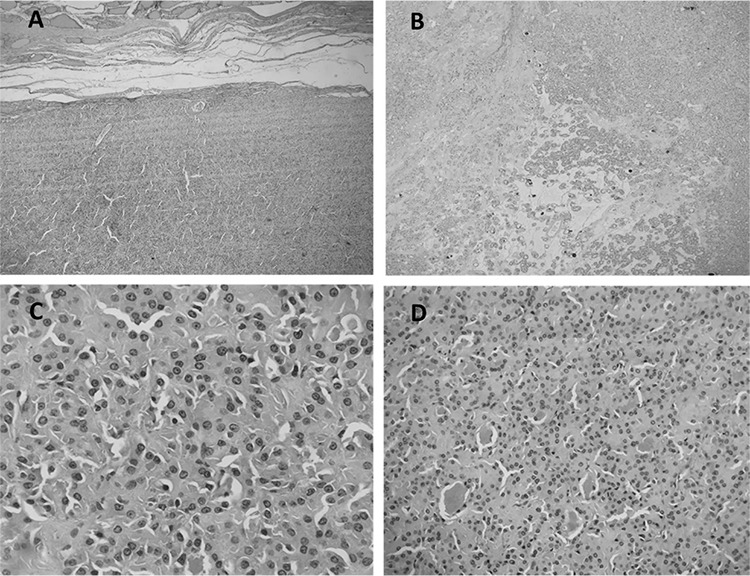
Histopathology evaluation revealed Hurthle cell adenoma (2.5 cm). Figure 2 displays histopathological characteristics of a well circumscribed neoplasm with a thin walled capsule (A). The low power view of adenoma at 4x (B) shows the cells arranged in sheets. Individual cells have abundant granular oncocytic cytoplasm with round large nuclei and prominent nucleoli (C, D). Thyroid incidentalomas are frequently detected on FDG PET/CT scan during oncological work-up. A normal thyroid gland shows homogenous, mild FDG uptake on PET/CT images (1). High, focal metabolic activity usually has a higher tendency to be malignant (2). In the absence of frank local invasion or adenopathy, it is difficult to characterize a thyroid nodule on CT scan (3). Heterogeneous complex textured thyroid nodules with an average size of 2.0 cm are radiologically concerning for malignancy (4). At our institution, the prevalence of thyroid incidentalomas identified by FDG PET/CT is 1.7% (5), which is in line with the prior literature (2). FDG uptake in a lesion is dependent on the rate of glycolysis. Malignant lesions take up FDG in significantly higher fractions as compared to benign lesions. Focal FDG uptake in the thyroid has a reported malignancy rate ranging from 24 to 74% (6,7,8). Diffuse increased thyroid uptake is usually related to benign causes such as Graves’ disease and thyroiditis (2). Our institutional experience has shown 48% of the focal FDG avid thyroid incidentalomas to be malignant. The case presented herein had a complex thyroid nodule and the appearance was concerning for metastatic involvement. Despite the higher clinical suspicion of malignancy, our final histopathology was consistent with a benign pathology, i.e Hurthle cell adenoma. Hurthle cells are oxyphillic variant of follicular cells, and depict granular cytoplasm due to the high content of intra-cytoplasmic mitochondria (9). The intense metabolic activity in this nodule on FDG PET/CT scan can be explained on the basis of this abundance of intra-cytoplasmic mitochondria. Most prior studies suggest that high SUV is associated with thyroid malignancy. However, some conflicting results have also been reported with overlapping SUV of benign and malignant thyroid incidentalomas (10). It has been reported that 1.5–2.1% of benign thyroid nodules might show high SUV in FDG PET/CT scans (11). In view of these false positive findings, high metabolic activity on FDG PET/CT cannot accurately distinguish between benign and malignant nodules. Our case is an example where intense avidity of a thyroid nodule on FDG PET/CT was deceptive for being a malignant lesion.

## References

[1] Liu Y (2009). Clinical significance of thyroid uptake on F18-fluorodeoxyglucose positron emission tomography. Ann Nucl Med.

[2] Nakamoto Y, Tatsumi M, Hammoud D, Cohade C, Osman MM, Wahl RL (2005). Normal FDG distribution patterns in the head and neck: PET/CT evaluation. Radiology.

[3] Hoang JK, Raduazo P, Yousem DM, Eastwood JD (2012). What to do with incidental thyroid nodules on imaging? An approach for the radiologist. Semin Ultrasound CT MR.

[4] Cohen MS, Arslan N, Dehdashti F, Doherty GM, Lairmore TC, Brunt LM, Moley JF (2001). Risk of malignancy in thyroid incidentalomas identified by fluorodeoxyglucose- positron emission tomography. Surgery.

[5] Hassan A, Riaz S, Zafar W (2016). Fluorine-18 fluorodeoxyglucose avid thyroid incidentalomas on PET/CT scan in cancer patients: how sinister are they?. Nuc Med Commun.

[6] Kim H, Kim SJ, Kim I, Kim K (2013). Thyroid incidentalomas on FDG PET/CT in patients with non-thyroid cancer - a large retrospective monocentric study. Onkologie.

[7] Kim TY, Kim WB, Ryu JS, Gong G, Hong SJ, Shong YK (2005). 18F-fluorodeoxyglucose uptake in thyroid from positron emission tomogram (PET) for evaluation in cancer patients: high prevalence of malignancy in thyroid PET incidentaloma. Laryngoscope.

[8] Are C, Hsu JF, Schoder H, Shah JP, Larson SM, Shaha AR (2007). FDG-PET detected thyroid incidentalomas: need for further investigation?. Ann Surg Oncol.

[9] Maximo V, Sobrinho-Simoes M (2000). Hurthle cell tumours of the thyroid. A review with emphasis on mitochondrial abnormalities with clinical relevance. Virchows Arch.

[10] Bertagna F, Treglia G, Piccardo A, Giubbini R (2012). Diagnostic and clinical significance of F-18-FDG-PET/CT thyroid incidentalomas. J Clin Endocrinol Metab.

[11] Pathak KA, Klonisch T, Nason RW, Leslie WD (2016). FDG-PET characteristics of Hürthle cell and follicular adenomas. Ann Nucl Med.

